# Apigenin, by activating p53 and inhibiting STAT3, modulates the balance between pro-apoptotic and pro-survival pathways to induce PEL cell death

**DOI:** 10.1186/s13046-017-0632-z

**Published:** 2017-11-28

**Authors:** Marisa Granato, Maria Saveria Gilardini Montani, Roberta Santarelli, Gabriella D’Orazi, Alberto Faggioni, Mara Cirone

**Affiliations:** 1 Department of Experimental Medicine, “Sapienza” University of Rome, Viale Regina Elena 324, 00161 Rome, Italy; 2 Department of Research, Advanced Diagnostics, and Technological Innovation, Regina Elena National Cancer Institute, 00144 Rome, Italy; 3 Department of Medical, Oral and Biotechnological Sciences, Tumor Biology Section, University ‘G. d’Annunzio’, Chieti, Italy

**Keywords:** Apigenin, Apoptosis, Autophagy, KSHV, p53, PEL, STAT3, vFLIP

## Abstract

**Background:**

Apigenin is a flavonoid widely distributed in plant kingdom that exerts cytotoxic effects against a variety of solid and haematological cancers. In this study, we investigated the effect of apigenin against primary effusion lymphoma (PEL), a KSHV-associated B cell lymphoma characterized by a very aggressive behavior, displaying constitutive activation of STAT3 as well as of other oncogenic pathways and harboring wtp53.

**Methods:**

Cell death was assessed by trypan blue exclusion assay, FACS analysis as well as by biochemical studies. The latter were also utilized to detect the occurrence of autophagy and the molecular mechanisms leading to the activation of both processes by apigenin. FACS analysis was used to measure the intracellular ROS utilizing DCFDA.

**Results:**

We show that apigenin induced PEL cell death and autophagy along with reduction of intracellular ROS. Mechanistically, apigenin activated p53 that induced catalase, a ROS scavenger enzyme, and inhibited STAT3, the most important pro-survival pathway in PEL, as assessed by p53 silencing. On the other hand, STAT3 inhibition by apigenin resulted in p53 activation, since STAT3 negatively influences p53 activity, highlighting a regulatory loop between these two pathways that modulates PEL cell death/survival.

**Conclusion:**

The findings of this study demonstrate that apigenin may modulate pro-apoptotic and pro-survival pathways representing a valid therapeutic strategy against PEL.

## Background

Apigenin is one of the major flavonoids being present in a variety of natural sources such as parsley, chamomile, celery, artichokes, and oregano [1]. Several studies have reported that apigenin can be used to prevent or successfully control tumor progression in vivo*,* in several animal models [2]. These effects have been shown to occur either through the activation of the p53 oncosuppressor gene [2, 3] or the inhibition of oncogenic pathways, such as NFkB [4], HIF1alfa [5] and AKT [6]. The latter has been reported to be a target of apigenin in Primary Effusion Lymphoma (PEL) cells [7]. PEL is a Kaposi Sarcoma Associated Herpesvirus (KSHV)-associated malignant B cell lymphoma highly refractory to conventional chemotherapies [8], but displaying a good susceptibility to treatment with natural products such as capsaicin [9, 10]. These molecules share the characteristic to concomitantly inhibit multiple oncogenic pathways such as AKT and STAT3, strongly involved in PEL cell survival [11–13]. The latter indeed positively regulates the expression of pro-survival molecules such as survivin, cyclinD1 and c-myc [14, 15] or anti-apoptotic proteins such as c-FLIP [16, 17].

Similarly to other flavonoids, apigenin has been reported to target and to inhibit STAT3 in several cancers [18, 19] sometimes concomitantly with the activation of p53 tumor suppressor functions [18], leading to reduction of cancer cell survival. Interestingly, it has been described that activated STAT3 may inhibit p53, repressing its pro-apoptotic activity [20] and that, on the other hand, wtp53 activation may reduce STAT3 tyrosine phosphorylation and interfere with its DNA binding activity in prostate cancer cell lines displaying constitutive STAT3 activation [21]. Furthermore, it has been reported that the loss of p53 function activates JAK2-STAT3 signaling to promote pancreatic tumor growth and gemcitabine resistance [22]. All these studies suggest a reciprocal influence between p53 and STAT3 in cancer. Based on this knowledge and on the reports showing that apigenin can target both STAT3 and p53 in cancer cells, in this study we investigated the effect of apigenin in PEL cells that display constitutive STAT3 activation and wtp53, and the underlying mechanisms [21]. ROS, highly produced in cancer cells including PEL, are essential for their survival since sustain the activation of STAT3 as well as of other oncogenic pathways. Intracellular ROS can be regulated by wtp53 [23] that, among its numerous activities, may reduce the intracellular ROS by up-regulating catalase, one of the most important ROS scavenger [24] or other anti-oxidant enzymes [25]. Thus, we next investigated whether ROS reduction by apigenin could lead to STAT3 de-phosphorylation and whether STAT3 inhibition could down-regulate the expression of downstream molecules such as c-myc and cyclin D1 that sustain cancer cell proliferation and/or affect cellular or viral anti-apoptotic molecules. Finally, we evaluated whether apigenin, previously reported to interfere with AKT activation, could also affect autophagy in PEL cells.

## Methods

### Cell culture and reagents

BC3 (American Type Culture Collection, Manassas, Va, USA; ATCC) and BCBL1 (kindly provided by Prof. P. Monini, National AIDS Center, Istituto Superiore di Sanità, Rome, Italy) are human B-cell lines infected by KSHV, established from patients affected by Primary Effusion Lymphoma (PEL). Primary B lymphocytes were obtained from healthy donors as previously described [26]. Cells were cultured in RPMI 1640 (Thermo Fisher Scientific, Waltham, MA, USA; 21,870) supplemented with 10% Fetal Bovine Serum (FBS) (Corning, NY, USA; 35-079), with L-glutamine and with streptomycin (100 μg/ml) (Corning, NY, USA; 30-002) and with penicillin (100 U/ml) (Corning, NY, USA; 25-005) in 5% CO_2_ at 37 °C.

In dose-dependent studies, BC3 and BCBL-1 cell lines were treated with apigenin (Api) (Sigma Aldrich, St Louis. MO, USA; 520-36-5) at 12.5 μM and 25 μM or with N-acetyl-L-cysteine (NAC) at 25 and 50μM (Sigma Aldrich, St Louis. MO, USA; 616-91-1), for 24 h. PEL cells were also cultured in a complete medium supplemented with AG490 (50 μM) (Calbiochem, Billerica, MA, USA; 658,411) for 24 h.

In order to investigate autophagy, cells were cultured in a complete medium with apigenin (12.5 μM) and after 24 h were treated with chloroquine (CQ) (10 μM) (Santa Cruz Biotechnology Inc., Heidelberg, Germany; sc-201,550), an inhibitor of vacuolar-H^+^-ATPase, for the last four hours.

### Cell viability

BC3, BCBL1 and B cells were plated in 12-well plates at a density of 8 × 10^5^ cells/well. Cells were treated in a dose-dependent manner with apigenin (Sigma Aldrich, St Louis. MO, USA; D150659;) at 12.5 μM and 25 μM or with NAC at 25 μM and 50μM (Sigma Aldrich, St Louis. MO, USA; 616-91-1), for 24 h. BC3 cells were also transiently transfected with empty [27] vector or si-p53 plasmid [28] as described below, and then treated with apigenin (12.5 μM) for 24 h.

A trypan blue (Sigma Aldrich, St Louis. MO, USA; 72,571) exclusion assay was performed to test cell viability. Live cells were counted by light microscopy using a Neubauer hemocytometer. The experiments were performed in triplicate and at least repeated three times.

### Measurement of intracellular reactive oxygen species (ROS) production

To measure reactive oxygen species (ROS) production, the 2′,7′-dichlorofluorescein diacetate (DCFDA) (Thermo Fisher Scientific, Waltham, MA, USA; D399) was used. DCFDA is a fluorogenic dye that, after diffusion in to the cell, is oxidized by ROS into 2′,7′-dichlorofluorescein (DCF), a highly fluorescent compound which can be detected by fluorescence spectroscopy. To measure ROS production, BC3 and BCBL1 cells were treated with apigenin at 12.5 μM and 25 μM for 24 h. Then, cells were washed with pre-warmed 1X PBS and were incubated at 37 °C with 10 μM DCFDA for 15 min in PBS. Subsequently, PEL cells were washed and analyzed in FL-1 by a FACScalibur flow cytometer (BD, USA), using CELLQuest software (BD Biosciences, San Jose, CA, USA). Live cells were gated according to their forward scatter (FSC) and side scatter (SSC) properties. For each analysis 10.000 events were recorded [29].

### Sub-G1 cell cycle analysis

For cell cycle analysis, the DNA content was analyzed using the method of Propidium Iodide (Sigma Aldrich, St Louis. MO, USA; P4170) staining and flow cytometry. BC3 and BCBL1 untreated or treated with apigenin at 12.5 μM and at 25 μM for 24 h. 5 × 10^5^ cells were washed with cold 1X PBS and fixed in 70% ethanol on ice for at least 1 h. Cell pellet was washed three times with cold 1X PBS and stained with 50 μg/ml PI for 15 min at 37 °C. Then, DNA content was measured by a BD Biosciences FACSCalibur. Cell debris was excluded from analysis by increasing the forward scatter threshold. Cells with a DNA content lower and a Side Scatter higher than that of G0/G1 cells, were considered as apoptotic cells, sub-G1. Data are representative of at least three independent experiments.

### Western blot analysis

1 × 10^6^ cells were washed twice with 1X PBS solution and centrifuged at 1500 rpm for 5 min. The pellet was lysed in a RIPA buffer containing 150 mM NaCl, 1% NP-40, 50 mM Tris-HCl (pH 8), 0.5% deoxycholic acid, 0.1% SDS, protease and phosphatase inhibitors. 20 μg of protein lysates were subjected to protein electrophoresis on 4-12% NuPage Bis-Tris gels (Sigma Aldrich, St Louis. MO, USA; N00322BOX), according to the manufacturer’s instruction. Then, the gels were blotted onto nitrocellulose membrane (Biorad, Milan, Italy; 162-0115) for 2 h in Tris-Glycine buffer. The membranes were blocked in PBS 0.1% Tween20 solution containing 3% of BSA, probed with specific antibodies and developed using ECL Blotting Substrate (Advansta, Menlo Park, CA, USA; K-12045-D20).

### Antibodies

In western blotting analysis, we used the following primary antibodies: rabbit polyclonal anti-PARP (1:500) (Cell Signaling, Danvers, MA, USA; 9542), rabbit polyclonal anti p21 (1:100) (Santa Cruz Biotech, Heidelberg, Germany; sc-397), mouse monoclonal anti-p53 (1:500) (Santa Cruz Biotech, Heidelberg, Germany; sc-126), mouse monoclonal anti-catalase (1:100) (Santa Cruz Biotec, Heidelberg, Germany; sc-271,803), mouse monoclonal anti-STAT3 (1:1000) (BD Transduction Laboratories, New Jersey, USA; 610,189), mouse monoclonal anti-phospho-STAT3 (p-Tyr705) (1:100) (Santa Cruz Biotec, Heidelberg, Germany; sc-8059), rabbit polyclonal anti-FLIP (1:100) (Santa Cruz Biotec, Heidelberg, Germany; sc-8347), rabbit polyclonal anti-cMyc (1:500) (Cell Signaling, Danvers, MA, USA; 5605) and rat polyclonal anti-vFLIP (1:10)(4C1) (kindly provided by Prof. Regina Feederle) [30]. To study autophagy, we used rabbit polyclonal anti-LC3 (1:1000) (Novus Biologicals, Cambridge, UK; NB100-2220SS) and mouse monoclonal anti-p62 (1:500) (BD Transduction Laboratories, New Jersey, USA; 610,883) antibodies.

Mouse monoclonal anti-β-actin (1:10,000) (Sigma Aldrich, St Louis. MO, USA; A5441) (1:10,000) was used as loading control. The goat polyclonal anti-mouse IgG-Horseradish Peroxidase Santa Cruz Biotechnology Inc., Heidelberg, Germany; sc-2005) and anti-rabbit IgG-HRP (Santa Cruz Biotechnology Inc., Heidelberg, Germany; sc-2004) were used as secondary antibodies. All the primary and secondary antibodies were diluted in PBS-0.1% Tween20 solution containing 3% of BSA (SERVA, Reno, NV, USA; 11,943.03).

### p53 silencing

BC3 cells, diluted in complete medium without antibiotics, were plated at a density of 3 × 10^5^ cells/well in 12 wells plates. Then, cells were transfected with empty vector [27] and si-p53 plasmid [28] using Lipofectamine 2000 (Thermo Fisher Scientific, Waltham, MA, USA; 1,880,845) for 48 h, according to the manufacturer’s instructions. Finally, BC3 cells were treated with apigenin (12.5 μM) for the last 24 h and then centrifuged at 1500 rmp at 4C°. Cellular pellets were lysed and protein extracts were subjected to electrophoresis, as described above.

### Densitometric analysis

The quantification of proteins bands was performed by densitometric analysis using the Image J software, downloaded from NIH web site (http://imagej.nih.gov).

### Statistical analysis

Results are represented by the mean ± standard deviation (SD) of at least three independent experiments and a two-tailed Student’s t-test was used to demonstrate statistical significance. Difference was considered as statistically significant when *p*-value was at least <0.05.

## Results

### Apigenin induces apoptosis and autophagy in PEL cells

**Fig. 1:**
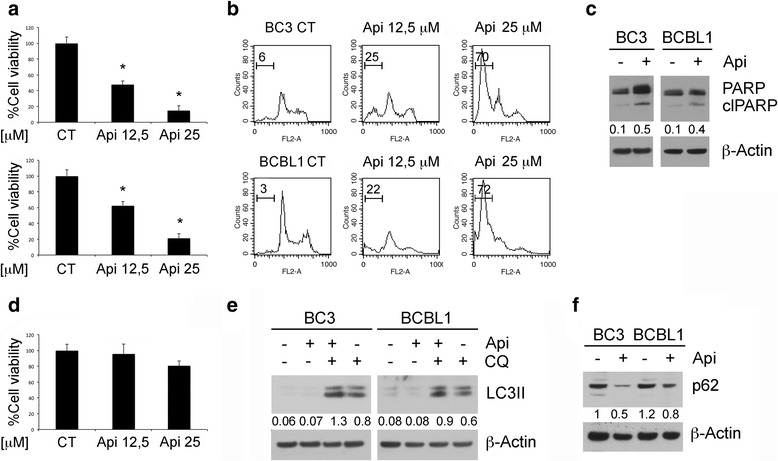
Apigenin induces apoptotic cell death and autophagy in PEL cell lines. BC3 and BCBL1 cell were treated with apigenin at the indicated concentrations (12,5 and 25 μM) for 24 h. **a** Cell survival was assessed by trypan blue exclusion assay. The histograms represent the mean of the percentage of cell survival plus SD of at least three independent experiments. _*_p< 0.05. **b** DNA content of PEL cell lines treated with apigenin (12,5 and 25 μM) for 24 h as measured with Propidium Iodide staining and analyzed by flow cytometry. The percentage of sub-G1 events is reported. FACS plots are representative of at least three independent experiments. Means plus SD of three independent experiments are: BC3 CT 5 ± 0.7, BC3 Api 12,5 28 ± 4, BC3 Api 25 73 ± 7; BCBL1 CT 3 ± 1, BCBL1 Api 12,5 24 ± 4, BCBL1 Api 25 75 ± 9; **c** The expression levels PARP uncleaved and cleaved (clPARP) were assessed by western blot analysis. β-Actin was used as loading control. Numbers are calculated by quantitative densitometric analysis and indicate the ratio of clPARP versus β-Actin. **d** Cell survival of primary B lymphocytes as assessed by trypan blue exclusion assay. The histograms represent the mean of the percentage of cell survival plus SD of at least three independent experiments. _*_p< 0.05. **e** PEL cells were treated with apigenin at 12,5 μM or chloroquine (CQ) at 10 nM and with both of them for 24 h and LC3-I/II expression was evaluated by western blotting. Numbers are calculated by quantitative densitometric analysis and indicate the ratio of LC3-II versus β-Actin. **f** BC3 and BCBL1 were treated with apigenin and p62 expression was evaluated by western blotting. Numbers are calculated by quantitative densitometric analysis and indicate the ratio of p62 versus β-Actin. Data are representative of at least three independent experiments

BC3, BCBL-1 PEL cell lines and B cells were treated with two different concentration of apigenin (12.5 and 25 μM) for 24 h. We found that apigenin was able to reduce PEL cell survival in a dose dependent fashion while it slightly affected B cell survival, as indicated by the trypan-blue exclusion assay (Fig. 1a and d). To evaluate the type of cell death induced by apigenin, the appearance of sub-G1 events was assessed by FACS analysis. The increased percentage of sub-G1 events in cells treated with different doses of apigenin (Fig. 1b) suggests the occurrence of apoptotic cell death, confirmed by the cleavage of PARP observed by western blot analysis (Fig. 1c). As autophagy is usually up-regulated in cancer cells undergoing cytotoxic treatments, we investigated whether apigenin could affect autophagy in PEL cells. We found that LC3-II expression increased in PEL cells treated with apigenin in presence of cloroquine in comparison with control cells (Fig. 1e), indicating the induction of a complete autophagic flux, further confirmed by the reduction of p62 (Fig. 1f), molecule mainly degraded through autophagy.

### Apigenin induced cell death correlates with the reduction of intracellular ROS

**Fig. 2:**
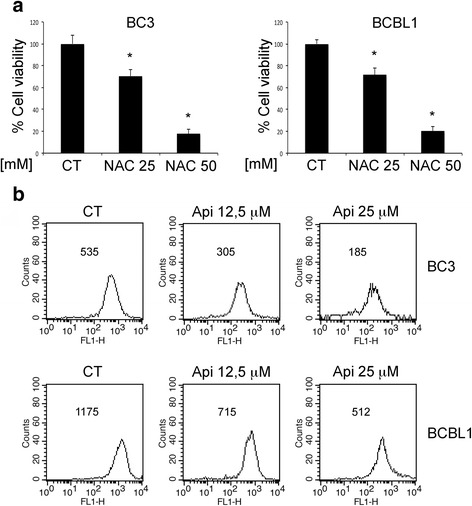
Apigenin reduces intracellular ROS. **a** BC3 and BCBL1 cells were treated with NAC at 25 and 50 mM for 24 h. Cell survival was assessed by trypan blue exclusion assay. The histograms represent the mean of the percentage of cell survival plus SD of at least three independent experiments. _*_p< 0.05. **b** PEL cell were treated with apigenin at 12,5 and 25 μM for 24 h. Intracellular ROS were evaluated by FACS analysis using DCFDA staining FACS plots are representative of three independent experiments*.* Numbers indicate the mean of fluorescence intensity. Means plus SD of three independent experiments are: BC3 CT 540 ± 31, BC3 Api 12,5 μM 313 ± 21, BC3 Api 25 μM 193 ± 20; BCBL1 CT 1185 ± 90, BCBL1 Api 12,5 μM 724 ± 52, BCBL1 Api 25 μM 525 ± 33

Intracellular ROS may sustain the activation of pro-survival pathways from which PEL cells are strongly dependent [13, 26]. To corroborate this finding, we treated PEL cells with the ROS scavenger N-acetylcisteine (NAC) at two different doses (25 and 50 mM) and found that it reduced cell survival in a dose-dependent fashion (Fig. 2a), Consequently, we investigated whether apigenin could affect the levels of intracellular ROS in PEL cells. As shown in Fig. 2b, apigenin caused a dose-dependent ROS decrease in both BC3 and BCBL-1 cells that correlated with the reduction of cell survival seen above (Fig. 1a and b) and similarly to the effect achieved by NAC (see above). These findings demonstrate the importance of ROS in the maintenance of PEL cell survival and that apigenin, similarly to NAC, can target it.

### Apigenin activates p53 up-regulating p21 and catalase expression in PEL cells

**Fig. 3:**
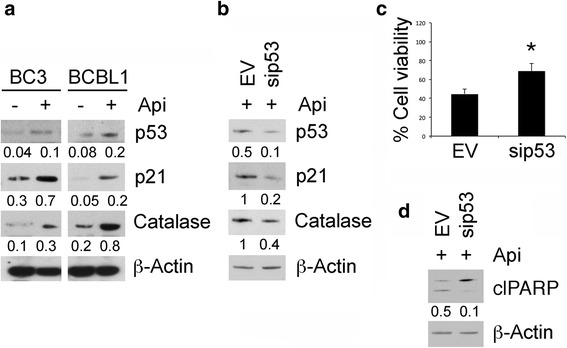
Apigenin activates p53 leading to an increase of catalase expression. **a** BC3 and BCBL1 cell lines were treated with apigenin 25μM for 24 h. p53, p21 and catalase expression was assessed by western blotting analysis. β-Actin was used as loading control. Numbers are calculated by quantitative densitometric analysis and indicate the ratio of specific proteins versus β-Actin. Data are representative of at least three independent experiments. **b** BC3 cells were transiently transfected with si-p53 vector to silence p53 protein for 48 h and treated with apigenin 25 μM for the last 24 h. p53, p21 and catalase expression was evaluated by western blotting. Numbers are calculated by quantitative densitometric analysis and indicate the ratio of specific proteins versus β-Actin. Data are representative of at least three independent experiments. **c** p53-silenced or control BC3 cell survival was assessed by trypan blue exclusion assay. The histograms represent the mean of the percentage of cell survival plus SD of at least three independent experiments. _*_p< 0.05. **d** The expression levels PARP uncleaved and cleaved (_Cl_PARP) were assessed by western blot analysis in p53-silenced or control BC3 cells. β-Actin was used as loading control. Numbers are calculated by quantitative densitometric analysis and indicate the ratio of clPARP versus β-Actin

Apigenin has been reported to activate p53 oncosuppressor function in wild-type (wt)- or mutant 53-carrying cancer cells [31, 32]. Therefore, here we evaluated whether apigenin could activate p53 in PEL. We found that apigenin treatment in BC3 and BCBL-1 up-regulated the levels of p21, target of p53, in comparison to the control cells (Fig. 3a); moreover, we also found that apigenin increased protein levels of catalase (Fig. 3a), one of the most important ROS scavenger enzymes reported to be activated by p53 [24, 33]. Next, p53 silencing in BC3 cells impaired both p21 and catalase up-regulation following apigenin treatment (Fig. 3b) and partially prevented apigenin-induced cell death as shown by viability and PARP cleavage (Fig. 3c and d). Altogether these results indicate that apigenin activated p53 to induce PEL cell death likely through reduction of intracellular ROS production by catalase.

### Apigenin, by diminishing ROS, reduces STAT3 activity

**Fig. 4:**
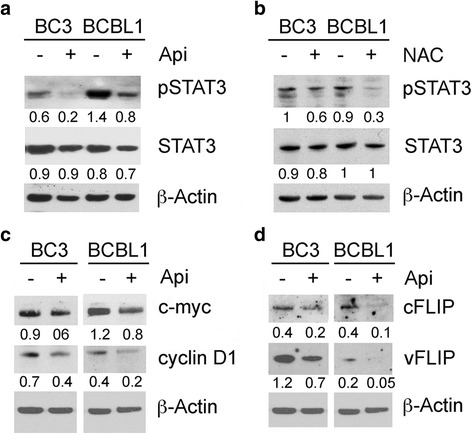
Apigenin de-regulating STAT3 activation, similarly to NAC, reduces the expression of pro-survival molecules c-myc, cyclin D1, cFLIP and vFLIP. BC3 and BCBL1 were treated **a** with apigenin at 25 μM or **b** with NAC at 25 mM for 24 h and pSTAT3 (Tyr 705) and total STAT3 expression were assessed by western blotting. β-Actin was used as loading control. Numbers are calculated by quantitative densitometric analysis and indicate the ratio of phosphorylated (p-STAT3) versus total protein (STAT3) and total protein (STAT3) protein versus β-Actin. **c** c-myc and cyclinD1 and **d** cFLIP and vFLIP expression was assessed by western blot analysis in PEL cells treated with apigenin at 25 μM for 24 h. Numbers are calculated by quantitative densitometric analysis and indicate the ratio of specific proteins (c-myc, cyclin D1, cFLIP and vFLIP) versus β-Actin. Data are representative of at least three independent experiments

Intracellular ROS are important to maintain cancer cell survival through the activation of oncogenic pathways such as STAT3 [33] that is strongly involved in PEL cell survival [11–13]. Therefore we evaluated whether apigenin could modify STAT3 activation and found that it strongly reduced STAT3 phosphorylation in both BC3 and BCBL1 cells (Fig. 4a), similarly to the effect achieved by the ROS scavenger NAC (Fig. 4b).

STAT3 is an important transcription factor that regulates the expression of a variety of pro-survival molecules, including c-myc and cyclin D1 [14]. Based on this knowledge we found that apigenin reduced c-myc and cyclin D1 levels in PEL cells (Fig. 4c). Then, we investigated if, besides molecules involved in cell proliferation, apigenin could also affect cellular or viral molecules that negatively regulate apoptosis. As shown in Fig. 4d, the expression of cellular (c)-FLIP, also regulated by STAT3 [34], and viral (v)-FLIP, important anti-apoptotic molecule, were reduced following apigenin treatment. Altogether, these findings suggest that apigenin, by targeting STAT3, could modulate pro-survival and anti-apoptotic pathways mainly regulated by STAT3 to achieve cell death in PEL.

### p53 activation by apigenin is involved in STAT3 dephosphorylation and STAT3 inhibition reduces its negative influence on p53

**Fig. 5:**
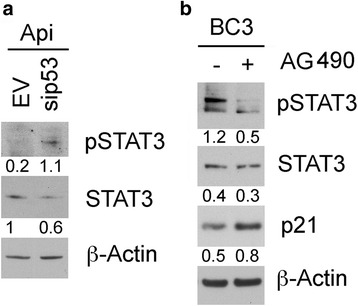
Down-regulation of p53 reduces apigenin-mediated STAT3 inhibition that in turn activates p53/p21. **a** BC3 cells were p53-silenced as described in Fig. 3b. Western blotting analysis was used to assess pSTAT3 (Tyr 705) expression. Numbers are calculated by quantitative densitometric analysis and indicate the ratio of phosphorylated (p-STAT3) versus total protein (STAT3) and total protein (STAT3) versus β-Actin. Data are representative of at least three independent experiments. **b** BC3 cells were treated with AG490 (50 μM) for 24 h and pSTAT3 (Tyr 705), total STAT3 and p21 expression was investigated by western blot. Numbers are calculated by quantitative densitometric analysis and indicate the ratio of phosphorylated (p-STAT3) versus total protein (STAT3) and total protein (STAT3) or p21 versus β-Actin. Data are representative of at least three independent experiments

As apigenin activated p53 and inhibited STAT3, we next evaluated whether a cross-talk existed between these two pathways, by performing p53 silencing. We found that, compared to the empty vector (EV), p53 silencing (sip53) rescued STAT3 phosphorylation in apigenin-treated BC3 cells (Fig. 5a), confirming the role of p53 in STAT3 inhibition. Since it has been reported that constitutively activated STAT3 can negatively influence p53 activity [20], we investigated whether the pharmacological inhibition of STAT3 by AG490 could in turn induce p53 activation. As shown in Fig. 5b, the efficient reduction of STAT3 phosphorylation by AG490 treatment correlated with up-regulation of p21 levels indicative of p53 activation. Altogether, these findings highlight the occurrence of a cross-talk between p53 and STAT3 during apigenin treatment that modulates PEL cell survival.

## Discussion

**Fig. 6:**
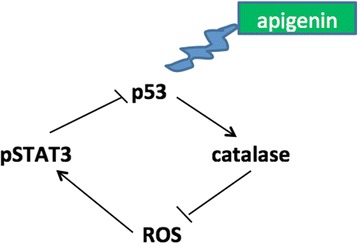
Schematic model of pathways activated or inhibited by apigenin

PEL, the KSHV-associated B cell lymphoma, is highly malignant and characterized by poor response to chemotherapies. In this study, we show that apigenin could represent a valid therapeutic strategy against PEL, also confirming a previous reported study [7]. Apigenin is a flavonoid widely distributed in plant kingdom displaying several beneficial effects also against cancer [3, 35–37]. We found that, among the underlying mechanisms leading to PEL cell death, apigenin induced a cross-talk between p53 and STAT3. Indeed apigenin induced the activation of p53, as revealed by p21 up-regulation, and induced catalase expression, reducing ROS, dephosphorylating STAT3 and preventing STAT3 inhibitory influence on p53 (Fig. 6). Although the influence of p53 on STAT3 [21] and of STAT3 on p53 [20], have been previously described, this study shows, for the first time, a cross-talk between the two pathways mediated by the reduction of intracellular ROS. p53 is strongly involved in the control of ROS level, since it can have both pro-oxidant and anti-oxidant properties that can be mediated by the inhibition or the activation of catalase activity [38]. The finding that apigenin, by activating the anti-oxidant response, reduced PEL cell survival is quite surprising, since it usually elevated oxidant species to induce cancer cell death [39]. However, it is well known that ROS levels need to be finely regulated since either their increase or their reduction reduce cell survival [23]. In one hand, high levels of ROS sustain oncogenic signaling, indeed PEL cells die when ROS decrease following treatment with metformin [26] or with NAC, while on the other hand ROS increase may also lead to cancer cell death, although cancer cells are equipped with high expression of molecules, such NRF2, that allow them to rapidly adapt to ROS increase.

STAT3, one of the pathway strongly sustained by ROS, is constitutively activated in PEL cells and plays a fundamental role for the survival of these cells, as well as of several other cancer cell types [13, 40]. It indeed may promote the transcription of a variety of pro-survival molecules such as survivin, c-myc and cyclin D1. Interestingly, STAT3 also interacts with AKT/mTOR pathway in PEL cells, indeed they can positively influence each other to promote cell survival and both are sustained by high levels of intracellular ROS [9, 10, 33]. On the other hand, ROS production is influenced by the expression of KHSV proteins such as v-FLIP [41], strongly down-regulated by apigenin, similarly to its cellular homologue c-FLIP. C-FLIP and v-FLIP are molecules involved either in the inhibition of apoptosis and of autophagy [42–44], therefore their down-regulation may contribute to the induction of both processes by apigenin in PEL cells. However, the inhibition of STAT3 is likely one of the main stimuli leading to autophagy induction by apigenin and indeed its activation, by regulating the release of cytokines by cancer cells [9] as well as by immune cells [45], may result in autophagy inhibition [46]. STAT3 activation and autophagy inhibition also correlated with an impairment of DC phenotypic and functional properties [45], besides interfering with other aspects of the immune response for which the autophagic process is also required [47]. Regarding cancer cells, autophagy activated by chemotherapies before or concomitantly with apoptosis can influence cell survival either positively, by helping cells to cope with basal or chemotherapy-induced stress, or negatively, by promoting the degradation of oncogenic molecules such as mutant p53 [48] or c-myc [10]. Furthermore, the effect of anti-cancer drugs on autophagy is worth to investigate, also beacause autophagy can promote the release of ATP [49] and activate the immune system and influence the overall survival of anti-cancer treatments [50]. Regarding this point, there are increasing evidences that the chemotherapy must not only be not toxic for the cells of the immune system, but it should also promote the exposure, or the release, of hidden molecules called damage-associated molecular patterns (DAMPs) that stimulate the immune response against cancer [51].

## Conclusions

In conclusion this study, showing the activation of a cross-talk between p53 and STAT3 by apigenin, unveils new molecular mechanisms through which this flavonoid leads to apoptosis and autophagy induction in PEL, an aggressive lymphoma harboring wt p53 and constitutive STAT3 activation, that is very difficult to successfully treat.

## Abbreviations

Api: Apigenin cFLIP Cellular FLICE-like inhibitory protein KSHV Kaposi’s sarcoma-associated herpesvirus LC3 Microtubule-associated protein 1A/1B-light chain 3 NAC N-acetylcysteine PEL Primary effusion lymphoma ROS Reactive oxygen species STAT3 Signal transducer and activator of transcription 3 v-FLIP viral-FLICE-Inhibitory protein
